# The Positive Side Effect of Anterior Cervical Decompression and Fusion on Axial Neck Pain

**DOI:** 10.1177/21925682241254036

**Published:** 2024-05-10

**Authors:** Andrea Redaelli, Pablo Bellosta-López, Francesco Langella, Paolo Lepori, Francesca Barile, Riccardo Cecchinato, Domenico Compagnone, Marco Damilano, Daniele Vanni, Claudio Lamartina, Pedro Berjano

**Affiliations:** 1 IRCCS Ospedale Galeazzi-Sant’Ambrogio, Milan, Italy; 2 202605 Universidad San Jorge, Villanueva de Gállego, Zaragoza, Spain; 3 DIBINEM Dipartimento di Scienze Biomediche e Neuromotorie, 46767 Università di Bologna, Bologna, Italy; 4 Department of Biomedical Sciences for Health, University of Milan, Milan, Italy

**Keywords:** neck pain, spinal diseases, spinal fusion, spine surgery

## Abstract

**Study Design:**

Observational Cohort Study.

**Objectives:**

This study aims to comprehensively assess the outcomes of anterior cervical spine surgery in patients who have undergone surgical intervention for radiculopathy or myelopathy, with a specific focus on the surgery’s impact on axial neck pain.

**Methods:**

Data from an institutional spine surgery registry were analyzed for patients who underwent anterior cervical spine surgery between January 2016 and March 2022. Patient demographics, clinical variables, and outcome measures, including the Neck Disability Index (NDI), numeric rating scales for neck and arm pain (NRS-Neck and NRS-Arm), and 36-Item Short Form Health Survey (SF-36) scores, were collected. Statistical analysis included paired t-tests, chi-squared tests, and multivariate linear regression.

**Results:**

Of 257 patients, 156 met the inclusion criteria. Patients showed significant improvement in NDI, NRS-Neck, NRS-Arm, SF-36 (Physical and Mental components), and all changes exceeded the minimum clinically important difference. Multivariate regression revealed that lower preoperative physical and mental component scores and higher preoperative NRS-Neck predicted worse NDI scores at follow-up.

**Conclusions:**

This study underscores that anterior cervical fusion not only effectively alleviates arm pain and disability but also has a positive impact on axial neck pain, which may not be the primary target of surgery. Our findings emphasize the potential benefits of surgical intervention when neck pain coexists with neurologic compression. This contribution adds to the growing body of evidence emphasizing the importance of precise diagnosis and patient selection. Future research, ideally focusing on patients with isolated neck pain, should further explore alternative surgical approaches to enhance treatment options.

## Introduction

Neck pain is one of the most frequent complaints in the musculoskeletal field, estimated to affect nearly 30% of the adult population.^1^ However, a precise definition of neck pain is lacking, making its real prevalence unclear. It is generally accepted that neck pain, in contrast with radicular pain, does not radiate to the arms and is situated between the scapula and shoulders, as well as between the occipital area and the inferior angle of the scapula.^2,3^

Radicular pain caused by cervical disc herniation or degenerative disc disease, unresponsive to conservative treatment, is often treated surgically with overall good results.^4^

Conversely, surgery is seldom employed for neck pain,^5^ as it commonly improves with nonoperative therapies, and identifying the source of pain is challenging due to the high prevalence of degenerative changes in asymptomatic subjects.^6^ Behavioral factors and psychological factors contributing to excessive muscular strain are also considered potential causes of neck pain.^7,8^

However, patients treated with surgery often experience a global improvement in symptoms, including not only radiating pain, but also axial neck pain,^9^ and occasionally even atypical symptoms such as headache and vertigo, frequently associated with cervical diseases.^10^

This study aims to comprehensively investigate the outcomes of cervical spine surgery in patients treated for radiculopathy or myelopathy, with a specific focus on evaluating its impact on axial neck pain. By delving deeper into these aspects, we aim to provide a more comprehensive understanding of the surgery’s “side effects” and potential benefits for patients. We hypothesize that the surgical procedure may play a significant role in reducing discogenic pain and the consequent resulting disability.

## Materials and Methods

### Participants

The study design utilized in this research is a retrospective observational cohort study. Data were extracted from the SpineReg institutional spine surgery registry, consisting of prospectively collected data between January 2016 and March 2022. The study was approved by the ethics committee of San Raffaele Hospital - Milan IRCCS (substantial amendment no. 3 of 05/09/2019 given the previous approval to the SPINEREG register with the number of the register of opinions of the ethics committee 93/INT/2015), and written consent was obtained from all patients. Inclusion criteria were adults over 18, proficient in Italian, underdoing cervical spine surgery via an anterior approach (Anterior Cervical Discectomy and Fusion -ACDF- or single level Anterior Cervical Corpectomy and Fusion -ACCF- for a maximum of 3 levels) with a diagnosis of radiculopathy or myelopathy not caused by infection, trauma, autoimmune diseases or tumors. Exclusion criteria included baseline Neck Disability Index (NDI) scores below 10/100, receiving cervical surgery with a posterior approach, and a follow-up period of less than 12 months. The longest follow-up measurement (range 12 to 72 months) was selected for analysis.

### Socio-Demographic and Clinical Variables

Socio-demographic and clinical data were collected at baseline, including age, sex, body mass index (BMI), smoking status, score in the physical status classification system according to the American Society of Anaesthesiologists (ASA), and the number of cervical levels treated.

### Outcome Measures

The primary outcome was the NDI, while secondary outcomes comprised of a numeric rating scale for neck pain (NRS-neck) and arm pain (NRS-arm), and the physical and mental component summary (PCS and MCS) of the 36-Item Short Form Health Survey.

The NDI stands as the most widely employed self-report measure for assessing disability related to neck disorders.^11^ Comprised of 10 dimensions evaluated on a 6-point scale ranging from 0 (no disability) to 5 (full disability), the cumulative score across these dimensions divided by the maximum score and then multiplied by 100 is expressed as NDI%. Notably, this percentage can be categorized into distinct disability levels: 0 to 8 indicates ‘no disability/recovered’, 10 to 28 ‘mild disability’, 30 to 48 ‘moderate disability’, 50 to 68 ‘severe disability’, and 70 to 100 ‘complete disability’.^12^

Furthermore, evaluations of neck and arm pain intensity at rest were conducted using an 11-point numeric rating scale, where 0 indicated ‘no pain’ and 10 ‘the worst imaginable pain’.^13^

The 36-Item Short Form Health Survey, designed to gauge the quality of life, encompasses 8 different scales: (1) limitations in physical activities; (2) limitations in social activities; (3) limitations in usual role activities because of physical health problems; (4) bodily pain; (5) psychological distress and well-being; (6) limitations in usual role activities because of emotional problems; (7) energy and fatigue; and (8) general health perceptions. Scores in these domains can be consolidated into 2 overarching components: PCS and MCS, expressed as a percentage from 0 (worst health) to 100 (best health).^14^

The minimal clinically important difference (MCID) was used to detect the smallest change in a treatment outcome that is considered meaningful for patients. It reflects the minimum improvement in neck pain measures that patients view as clinically relevant. The MCID after cervical spine surgery is established at 7.5 for NDI, 4.1 for PCS, and 2.5 for NRS-neck and NRS-arm.^13^ While no defined threshold exists for the minimum clinically important difference in MCS for patients undergoing cervical spine surgery, it has been delineated between 3.2 and 6.8 by Badhiwala et al. study.^15^

### Statistical Analysis

Statistical analysis was performed using SPSS v.25 (IBM, Chicago, IL, USA), and statistical significance was set at *P* < .05.^16^ Data distribution was assessed using the Kolmogorov-Smirnov test, and variables were reported as mean and standard deviation, median and interquartile range, or n and percentage as appropriate.

Paired *t* test was used to determine any difference between baseline and follow-up scores for all outcome measures. Cohen’s *d* was calculated to determine the magnitude of the change by the standardized difference between baseline and follow-up means, where *d* ≥ .2, *d* ≥ .5, and *d* ≥ .8 represent small, medium, and large effect sizes, respectively.^16^ Additionally, a Chi-squared test was conducted to compare the frequency of levels of disability according to the NDI (ie, ‘no disability/recovered’, ‘mild disability’, ‘moderate disability’, and ‘severe or complete disability’) at baseline and follow-up.

Finally, to control for potential baseline characteristics effects and to assess what measures interacted in predicting NDI at follow-up higher than 12 months post-surgery, a stepwise multivariate linear regression was conducted, including age, sex, BMI, smoking status, ASA >2, the number of cervical levels treated, and the length of the follow-up, as well as pre-operative NDI, NRS-Neck, NRS-Arm, PCS, and MCS as independent variables. Cohen’s *f*^2^ index was calculated to determine the magnitude of the contribution for each variable in the predicting model, where *f*^2^ ≥ .02, *f*^2^ ≥ .15, and *f*^2^ ≥ .35 represent small, medium, and large effect sizes, respectively.^16^

## Results

After inclusion/exclusion criteria 156 patients were enrolled in this study. Notably, 28 patients were excluded due to presenting baseline NDI scores lower than 10%, 73 patients due to undergoing cervical surgery with a posterior approach, and 79 due to lack of follow-up measurement of at least 12 months. The baseline characteristics of the 156 patients are provided in supplementary materials (Supplemental Material 1). The participants adhering to all eligibility criteria showed a lower rate of smokers and higher NRS-arm pain at baseline. The mean follow-up period for the included participants was 28 ± 17 months (range 12-60).

### Neck Disability Index

A significant reduction in the NDI score was observed at follow-up, with a mean difference of 18.4 (95%CI [15.0 to 21.8]; *P* < .001; *d* = .9) (Table 1).

**Table 1.** Mean Scores of Outcome Measures at Baseline and Follow-Up (n = 156).

	*Baseline*	*Follow-up*	*P*	*ES*
NDI (0-100)	38.6 ± 19.7	20.2 ± 19.5	<.001	0.9
NRS-neck (0-10)	5.6 ± 3.3	3.2 ± 3.1	<.001	0.8
NRS-arm (0-10)	6.8 ± 2.8	3.2 ± 3.2	<.001	1.2
MCS (100-0)	36.1 ± 8.1	42.4 ± 10.4	<.001	0.8
PCS (100-0)	43.2 ± 12.1	52.0 ± 10.8	<.001	0.7

Values are expressed in mean ± standard deviation or n (%). MCS: Mental Component Summary; NDI: Neck Disability Index; NRS: Numeric Rating Scale; PCS: Physical Component Summary. ES: Cohen’s *d* effect size.

This reduction in NDI was also evident in the frequency distributions of disability levels among baseline and follow-up measurements (*P* < .001) (Table 2).

**Table 2.** Patient Classification According to the Neck Disability Index at Baseline and Follow-Up.

	*Baseline NDI*	*Follow-up NDI*
Severe or complete disability	45 (29%)	15 (10%)
Moderate disability	52 (33%)	25 (16%)
Mild disability	59 (38%)	60 (39%)
No disability/Recovered	0 (0%)	56 (36%)

Values are expressed as frequency distributions of disability levels and the corresponding percentage (%). NDI: Neck disability index.

#### Numeric Rating Scale for Neck Pain and Arm Pain

At follow-up, a significant reduction in the NRS-Neck and NRS-Arm was noted, with a mean difference of 2.5 (95%CI [2.0 to 3.0]; *P* < .001; *d* = .8) and 3.6 (95%CI [3.0 to 4.2]; *P* < .001; *d* = 1.2), respectively (Table 1).

#### Physical Component Summary and Mental Component Summary

A significant improvement in the PCS and MCS was observed at follow-up, with a mean difference of −6.3 (95%CI [-9.2 to −3.5]; *P* < .001; *d* = .7) and −8.8 (95%CI [-12.2 to −5.5]; *P* < .001; *d* = .8), respectively (Table 1).

#### Regression Model for NDI Score at Follow-Up

In the multivariate linear regression, factors hierarchically found to significantly predict worse NDI score at follow-up included lower pre-operative PCS (Beta = −.81 95%CI [−1.17 to −.46]; *P* < .001; *f*^2^ = .15), lower pre-operative MCS (Beta = −.37 95%CI [−.61 to −.14]; *P* = .002; *f*^2^ = .08), and higher preoperative NRS-Neck (Beta = 1.29 95%CI [.46 to 2.13]; *P* = .003; *f*^2^ = .05). Pre-operative NDI, NRS-Arm, age, gender, BMI, being a smoker, ASA >2, number of cervical levels treated, and the follow-up length did not predict outcome.

## Discussion

Neck pain typically exhibits a positive response to conservative treatment and often improves over time. Conservative care includes manual therapy, pain medications, physical exercise and, in certain cases, more aggressive treatments such as epidural, neural root, or facet joint injections.^5^ Typically, conservative treatments can effectively manage neck pain without neurological impairment.^17^ Surgery is typically only considered for cases involving nerve root or spinal cord problems caused by a specific disc herniation or compression.^6,18^

In our retrospective observational cohort study, data from the SpineReg registry were analyzed, involving 156 patients who underwent anterior cervical spine surgery. The results of our findings demonstrated significant reductions in both neck disability and pain, underscoring the favorable outcomes in terms of disability alleviation, pain reduction, and enhanced quality of life following surgery.

The attribution of psycho-social issues as potential causes of neck pain stems more from the challenge of establishing a structural diagnosis than from an actual psychological disease. Given the high prevalence of disc disease in the asymptomatic population, pinpointing the exact source of pain often proves challenging.^19^ Consequently, surgical treatment for axial neck pain lacks robust scientific support and is generally discouraged.^5^ Nevertheless, it is common to observe improvement in the axial component of pain in patients undergoing surgery for cervical radiculopathy or myelopathy, motivating the focus of this study. In the literature, limited research has delved into surgical treatments for isolated neck pain. Early studies, such as Riley et al^20^ in 1969, reported 72% excellent or good results in 93 patients undergoing ACDF for axial neck pain. More recent studies, like Brigham and Tsahakis,^21^ reported 89% good outcomes in 36 subjects with both radicular and axial neck pain. Whitecloud and Seago^19^ collected 40 patients with pure axial neck pain confirmed by pre-operative discography who underwent ACDF: they had 32% excellent outcomes and 38% good outcomes, for an overall success of 70%. A similar study based on provocative discography on 27 patients was conducted by Siedenrock and Aebi^22^ who obtained good or excellent results in 73% of subjects. Patients undergoing two-level fusions reported more “good to excellent results” (85.7%) compared with those undergoing one-level fusion (61.9%). Patients experiencing radiating pain to the upper limbs exhibited a more favorable outcome compared to those without distinct radiating pain to the upper extremities preoperatively. Better results also were found in patients with pain onset after cervical spine trauma than those without. Finally, Palit et al^9^ in 1999 reported 79% personal satisfaction after ACDF for isolated neck pain in 38 patients. In this paper, the mean NRS for neck pain before surgery was 8.3 and became 4.1 after surgery. Similarly, the mean NDI decreased after the surgical treatment (58 vs 39). Our study, based on retrospectively analyzed but prospectively collected data, presents results comparable to those of these studies, with a significantly larger population.^23,24^ Although other studies in the literature report less favorable outcomes and success rates ranging from 30% to 46%,^22^ our study shows an improvement in NDI and SF-36 (both MCS and PCS) and a decrease in arm and neck pain when comparing baseline and final follow-up. The statistically significant improvement in all outcome measures, exceeding the minimum clinically important difference, demonstrates that anterior cervical fusion not only achieves its primary goal of reducing arm pain and improving disability but also improves axial neck pain, a secondary benefit not initially targeted by the surgical treatment. This improvement in axial neck pain can be defined as a positive collateral effect of anterior cervical decompression and fusion.

Neck pain caused by cervical disc herniation is probably due to impingement and irritation of fibres forming the posterior distribution of spinal nerves innervating cervical muscles. This may explain the positive response of neck pain to cervical decompression and fusion in the same way as radiating arm pain and paresthesia improve after ACDF and ACCF. However, it is essential to acknowledge that other causes of neck pain, such as tumours, infections, and fractures, necessitate completely different management and treatment approaches. While these pathologies are relatively rare compared to degenerative diseases, they demand prompt attention.^25^ Additionally, cervical spine deformities, such as kyphosis or scoliosis, can also contribute to neck pain.^26^

Moreover, it is crucial to recognize that non-spinal causes of neck pain may occur. Conditions such as Pancoast tumors, gallstones and other abdominal diseases can lead to radiating pain in the neck region.^27,28^ Issues in and around the shoulder joint may also manifest as neck pain near the shoulder.^3^

Historically, cervical spine surgery exclusively for isolated neck pain associated with most degenerative conditions, without neurologic compression, has exhibited a low overall success rate.^5^ Current guidelines on the management of neck pain discourage surgery as an option.^5,29^ Neck pain is highly prevalent and typically tends to improve spontaneously over time. The high prevalence of degenerative changes in the asymptomatic population, coupled with the difficulty in pinpointing the exact source of pain, supports the overall agreement with the discouragement of surgery for cervical pain.^5^ However, it is crucial to note that neurological compression in the cervical spine can cause isolated neck pain.^23^ In addition to neck pain alone, nerve compression is often (though not always) associated with other neurological symptoms, such as radiating pain down the arm, numbness, weakness, difficulty controlling the arms, or difficulty with gait and balance.^24^ The differentiation of pain arising from neurologic compression vs mechanical pain originating from spinal structures, is a critical step in evaluating a patient, as the treatment may differ. Precise diagnosis and identification of the type of neck pain are of paramount importance, as neck pain caused by neurological compression may necessitate and significantly benefit from cervical spine surgery.

A potential limitation of this study involves selection bias, as the analysis focused on patients affected by cervical radiculopathy or myelopathy and with some degrees of axial neck pain. It could be argued that good clinical outcomes in terms of neck pain are strictly related to the improvement of the patient’s main complaints, which were radiating symptoms. In particular, the amelioration of radiculopathy or myelopathy is posited to influence axial pain and its concomitant neck disability. It would be relevant to replicate a similar study on a population of subjects with isolated neck pain, as some authors did in the past, reporting satisfactory results.^9^ Moreover, the study exclusively considered patients who underwent cervical surgery with a single anterior approach. This choice aimed to avoid negative biases induced by the posterior cervical approach, which is generally considered more painful and associated with major complaints.^9,30^ The mean follow-up of this study was 28 ± 17 months, with the main variations in PROMs occurring during the first year after surgery.

## Conclusion

In conclusion, this observational study provides valuable insights into the effectiveness of surgical intervention for isolated neck pain stemming from cervical spine issues. The results challenge established norms by revealing that anterior cervical fusion surgery not only mitigates arm pain and disability but also brings about a noteworthy and significant improvement in axial neck pain. The positive outcomes, observed across diverse patient-centered measures, emphasize the potential advantages of surgical decompression, particularly in scenarios where neurologic compression contributes to neck pain. While acknowledging limitations such as selection bias, this study contributes to advancing our understanding of surgical approaches for neck pain and paves the way for further research into the mechanisms underlying these improvements. These findings carry substantial implications for clinical decision-making, advocating for a nuanced evaluation of surgical options when managing isolated neck pain with nerve impingement.

## Supplemental Material

Supplemental Material - The Positive Side Effect of Anterior Cervical Decompression and Fusion on Axial Neck Pain Supplemental Material for The Positive Side Effect of Anterior Cervical Decompression and Fusion on Axial Neck Pain by Andrea Redaelli, Pablo Bellosta-López, Francesco Langella, Paolo Lepori, Francesca Barile, Riccardo Cecchinato, Domenico Compagnone, Marco Damilano, Daniele Vanni, Claudio Lamartina, and Pedro Berjano in Global Spine Journal

## Data Availability

The datasets used and/or analyzed in the present study are available from the corresponding author upon reasonable request.*
