# Supplementary material for: The Positive Side Effect of Anterior Cervical Decompression and Fusion on Axial Neck Pain
Source: Global Spine J. 2024 May 10;15(3):1608–13. doi: 10.1177/21925682241254036 (PMC11572232; doi:10.1177/21925682241254036)
Supplement: Supplemental Material - The Positive Side Effect of Anterior Cervical Decompression and Fusion on Axial Neck Pain [file sj-pdf-1-gsj-10.1177_21925682241254036.pdf]

Supp 1. Baseline characteristics of participants included in the study compared with those excluded due to a shorter follow-up than 12 months.

	Included n=156	*Excluded* n=79	P
Age (years)	52.6 ± 11.3	53.7 ± 12.1	0.495
Female (n, %)	78 (50.0%)	36 (45.6%)	0.581
BMI (kg/m^2)	24.7 ± 4.2	24.3 ± 3.7	0.556
Smoker (n, %)	35 (22.4%)	31 (39.2%)	0.009
ASA ≥3 (n, %)	16 (10.3%)	9 (11.4%)	0.824
Cervical levels treated	2 [1-2]	2 [1-2]	0.291
NDI (0-100)	38.6 ± 19.7	40.1 ± 16.8	0.562
NRS-Neck (0-10)	5.6 ± 3.3	5.5 ± 3.2	0.854
NRS-Arm (0-10)	6.8 ± 2.8	5.8 ± 3.5	0.021
PCS (100-0)	36.1 ± 8.1	35.0 ± 7.4	0.349
MCS (100-0)	43.2 ± 12.1	47.7 ± 12.2	0.126

Values are expressed in mean ± standard deviation, median [interquartile range], or n (%). ASA: American Society of Anaesthesiologist class; BMI: Body Mass Index; MCS: Mental Component Summary; NDI: Neck Disability Index; NRS: Numeric Rating Scale; PCS: Physical Component Summary.
